# L-Ascorbic Acid 2-Phosphate-Releasing Poly(trimethylene carbonate) Increases Cell Proliferation and Collagen Production: A Novel Material for Pelvic Organ Prolapse Tissue Engineering

**DOI:** 10.1007/s13770-026-00831-3

**Published:** 2026-07-14

**Authors:** Katri Ala-Mononen, Dirk Grijpma, Alexander Efimov, Saana Mustakoski, Kirsi Kuismanen, Susanna Miettinen, Reetta Sartoneva

**Affiliations:** 1 Faculty of Medicine and Health Technology, Tampere University, Tampere, Finland; 2 Tays Research Services, Wellbeing Services County of Pirkanmaa, Tampere University Hospital, Tampere, Finland; 3 Advanced Organ Bioengineering and Therapeutics, Department of BioEngineering Technologies, Techmed Centre, University of Twente, Enschede, The Netherlands; 4 Faculty of Engineering and Naturals Sciences, Tampere University, Tampere, Finland; 5 Department of Obstetrics and Gynaecology, The Wellbeing Services County of Pirkanmaa, Tampere University Hospital, Tampere, Finland; 6 Department of Obstetrics and Gynaecology, The Wellbeing Services County of South Ostrobothnia, Seinäjoki, Finland

**Keywords:** L-ascorbic acid 2-phosphate, Poly(trimethylene carbonate), Tissue engineering, Pelvic organ prolapse, Collagen

## Abstract

**Background:**

Pelvic organ prolapse (POP) occurs due to the weakening of the pelvic floor connective tissues and their decreased collagen content, leading to the descent of pelvic organs into or through vagina. Therefore, biodegradable materials designed to enhance collagen production could be a future option for POP treatment.

**Methods:**

Here, we studied novel L-ascorbic acid 2-phosphate (A2P)-releasing biodegradable poly(trimethylene carbonate) (PTMC) materials. The effects of varying A2P concentrations (0, 5 or 10 wt%) were evaluated regarding material characteristics, such as component release and mechanical and surface properties. Further, materials’ effect on proliferation, collagen production and attachment of human vaginal stromal cells (hVSCs) and human adipose-derived stromal cells (hASCs) were analyzed during 14-day culture period.

**Results:**

A2P-releasing PTMC had good handling characteristics and mechanical properties, such as high tensile and suture retention strength. Rapid, yet controlled release of A2P from the PTMC was observed in *in vitro* release studies. Our results demonstrate that A2P-releasing PTMC significantly increased the proliferation and total collagen content of both hVSCs and hASCs. Further, genetic expression of collagen types I (COL I) and III (COL III) significantly increased on A2P-releasing PTMC membranes, and extracellular COL I fibers were visible in cultures on PTMC5. Increased attachment and cell sheet formation were observed in both hVSC and hASC cultures on A2P-releasing PTMC, and A2P increased α-smooth muscle actin (α-SMA) expression in hASCs.

**Conclusion:**

Our results indicate that A2P-releasing PTMC is a promising option for enhancing tissue regeneration in applications for POP tissue engineering.

## Introduction

Pelvic organ prolapse (POP) is a condition affecting up to 50% of women over the age of 50 [1]. It occurs when the pelvic floor's supportive structures weaken, leading to the descent of pelvic organs such as the bladder, uterus, or rectum into or even through the vagina [1, 2]. The most common symptoms associated with POP are vaginal bulging, urinary and defecation problems, and sexual dysfunctions. These symptoms can have a huge impact on women’s quality of life and may even increase the risk for anxiety and depression. [3, 4] Even up to 11–19% of women need surgical treatment for POP [1, 2]. Currently, the first-line surgical treatment of POP is native tissue repair, however, it has a high recurrence rate of up to 20–30%. [2, 5] Previously, non-absorbable polypropylene (PP) meshes have been used for relapsed POP, yet those meshes are associated with various complications, such as chronic pain and mesh exposure through vaginal tissues. Due to a high complication rate, PP meshes have been reclassified as a high-risk devices by the US Food and Drug Administration. [5] Therefore, safer and more efficient surgical treatment options are needed to address the increasing patient population.

Tissue engineering, combining biomaterials, cells, and active agents to promote tissue regeneration could offer an effective treatment option for POP. Especially in POP applications, it is important to develop strategies to enhance collagen production. Further, the biomaterial should have sufficient mechanical properties to withstand suturing and the mechanical forces in the pelvic floor. Poly(trimethylene carbonate) (PTMC) is a biodegradable and biocompatible polymer that has high elasticity and can withstand cyclic loading, which makes it a promising material for POP applications [6–8]. Further, PTMC erodes via enzymatic surface erosion without producing acidic degradation products [9]. It has been studied for various tissue engineering applications, such as vascular applications [10], and PTMC containing meshes for hernia repair have already been approved for clinical use [11].

The pelvic floor muscles, connective tissues, and ligaments are essential for sustaining pelvic organ support. The pelvic floor connective tissue is predominantly composed of vaginal stromal cells which are mainly fibroblasts, and extracellular matrix (ECM) components, such as collagen types I (COL I) and III (COL III) and elastin [12, 13]. Decreased levels of collagen and elastin in the pelvic floor connective tissues have been associated with POP [12–14]. To increase the ECM synthesis, ascorbic acid (AA) and its derivatives have been previously studied in combination with polylactic acid (PLA) and poly(l-lactide-co-ε-caprolactone) (PLCL) for POP tissue engineering [15–17]. Other compounds, such as basic fibroblast growth factor and estradiol have also been studied for collagen production enhancement [18, 19]. In this study, we used L-ascorbic acid 2-phosphate (A2P) which is a more stable derivative of AA and has been shown to increase the proliferation and collagen synthesis of various cell types [16, 17, 20, 21], making it highly appealing compound for enhancing connective tissue regeneration of the pelvic floor. Despite its potential, the benefits of combining A2P with PTMC have not yet been explored.

In this study, we aimed to demonstrate the potential of A2P-releasing PTMC membranes for POP tissue engineering. Especially, the aim was to investigate how A2P affects the collagen production, proliferation, and viability of human vaginal stromal cells (hVSCs) and human adipose-derived stromal cells (hASCs) on the PTMC membranes. Furthermore, the goal was to determine the release of A2P and its effect on the mechanical properties of PTMC.

## Methods

### Preparation of poly(trimethylene carbonate) membranes

High molecular weight PTMC was synthesized by ring-opening polymerization of trimethylene carbonate (Huizhou Foryou Medical Devices, China). A 2 wt% solution of PTMC in chloroform (Emparta, Germany) was prepared. Further, a dispersion of 2 wt% 2-phospho-L-ascorbic acid trisodium salt (A2P; Sigma-Aldrich, Germany) in chloroform was prepared by ultrasonic treatment. Thereafter, PTMC solutions containing 0, 5 or 10 wt% of dispersed A2P relative to PTMC (PTMC0, PTMC5 and PTMC10) were prepared by mixing. Solutions were then precipitated in isopropyl alcohol (Atlas and Assink Chemie B.V., Netherlands), dried in vacuum oven for 2–4 days and compression molded into 0.3 mm thick films at 130°C. Disk shaped specimens with a diameter of 11 mm were punched out of the films and sterilized by gamma irradiation under vacuum (25–50 kGy, STERIS, Etten-Leur, Netherlands).

### Mechanical testing

Tensile stress-strain experiments as well as suture retention strength (SRS) measurements were carried out in triplicate (n = 3) using a DMA 850 (TA Instruments, DE, USA). Specimens with dimensions approximately 15 × 2.5 × 0.3 mm^3^ were used in the tensile testing experiments and SRS measurements. The elastic modulus was determined from the initial slope of the stress-strain curve, and the yield stress from the point in the stress strain curve where the slope equals zero. Measurements were conducted at a test speed of 0.5 mm/min and preload of 0.01g was applied prior to measurement. In SRS measurements, a stainless-steel wire (0.1 mm diameter) was looped through the specimen approximately 2 mm from the top of the sample. The ends of the wire were clamped in the top grip of the DMA instrument, and the bottom of the sample was clamped in the bottom clip. The force to tear the sample was determined at a speed of 0.5 mm/min. The SRS was normalized to the thickness of the specimen.

### Water contact angle measurements

Contact angle measurements were conducted on dry PTMC membranes using Attension Theta Lite optical tensiometer (Biolin Scientific AB, Sweden) with sessile drop method. Measurement was conducted by placing 3 μl droplets of distilled water on the surface of the material and the contact angles from the left and right sides of the droplet were measured for 10 seconds. Five replicates with 6 to 8 droplets were measured for each material (n = 38–40).

### X-ray microtomography imaging

The 3D structure of PTMC5 and PTMC10 membranes (n = 3) were analyzed using X-ray microtomography (micro-CT). A Zeiss Xradia MicroXCT-400 device (Pleasanton, CA, USA) was operated at a tube voltage of 60 kV and a tube current of 166 µA. Two magnifications were used to obtain both an overall view and a more detailed view. A total of 1601 projections were acquired with pixel sizes of 22.78 µm and 2.28 µm, and exposure times of 4 and 7 seconds, respectively. Reconstruction was performed using XMReconstructor software. Image processing, particle size calculations, and visualizations were carried out using Avizo 3D 2023.1.1 software (Thermo Fisher Scientific, Waltham, MA, USA). Particles with diameter of one pixel (2.28 µm) were excluded from particle distribution calculations.

### Ascorbic acid 2-phosphate release from the membranes

A2P release from PTMC5 and PTMC10 membranes was measured with UV-Vis spectrophotometer. Four parallel samples (n = 4) of weight 22 ± 1 mg were incubated in 4 ml of phosphate buffered saline (PBS) or 20 µg/ml of cholesterol esterase (CE) in PBS at +37°C. Samples were removed and placed into fresh solution after 1, 2, 3, 4, 5, 6, 7 and 8 hours and 1, 2, 3, 6 and 7 days of incubation. For the samples incubated in PBS, measurement was made also on day 8. Incubation solutions were measured with UV-Vis spectrophotometer (Agilent Technologies, CA, USA) at 260 nm wavelength and released A2P was calculated using standard curve. Cumulative A2P release from PTMC5 and PTMC10 were calculated as a percentage of theoretical maximum of A2P in each material when assuming that materials had exactly 5 or 10 wt% of A2P, respectively.

### Cell isolation and culture

Isolation of hVSCs was done as previously described by Sartoneva *et al*. from vaginal tissue pieces obtained from three donors undergoing elective vaginectomy in Tampere University Hospital [22]. The hASCs were isolated from adipose tissues collected from three donors undergoing elective surgeries in Tampere University Hospital and hASC isolation was conducted as described previously by Kyllönen et al. [23]

Both hVSCs and hASCs used in this study were thawed and cultured in basic medium (BM) containing 5% human serum (Biowest, Nuaillé, France) and 1% of antibiotics (100 U/ml penicillin and 0.1 mg/ml streptomycin, Lonza, BioWhittaker, Verviers, Belgium) in Minimum Essential Medium Alpha (Gibco by Life Technologies) at +37°C in a humidified atmosphere with 5% of CO_2_ until confluent. The cells were used with the approval of the ethics committee of Pirkanmaa Hospital District, Tampere, Finland (hVSCs: R15051 and hASCs: R15161) and written consent from the donors.

### Cell seeding on membranes

Prior to cell seeding, PTMC membranes were fixed on the bottom of cell culture plates (Nunc, Thermo Fisher Scientific) with CellCrown 24NX (area: 0.58 cm^2^, Scaffdex, Tampere, Finland) inserts and incubated with BM at +37°C for 4 hours. After pre-wetting, medium was changed and 4000 hVSCs or 2000 hASCs were seeded on each membrane in three 10 µl droplets inside the insert. The cells were seeded in passage 4 for all analyses. Polystyrene (PS) wells (area: 1.9 cm^2^) were used as control samples. The cells were cultured at +37°C in a humidified atmosphere with 5% of CO_2_ for 1, 7 or 14 days depending on the analysis and culture medium was changed three times a week.

### Ascorbic acid 2-phosphate release in cell culture medium

A2P release was determined directly from the cell culture medium using ^31^P-NMR (phosphorus-31 nuclear magnetic resonance). The medium from hVSC cultures on PTMC5 and PTMC10 was collected at each medium change during the 14-day culture period with all donor lines (n = 3) and stored at -80°C. Before analyzing, samples were thawed at RT and medium samples from three biological replicates from each donor line were combined. Samples were spiked with 0.1 mg/ml sodium hypophosphite monohydrate (NaPO_2_H_2_·H_2_O; Sigma-Aldrich, 99% purity) in ultrapure water. The ^31^P-NMR measurements were done in Wilmad precision 5 mm NMR tubes, on a JEOL JNM-ECZ500R (11.747 T) spectrometer at 202.46 MHz equipped with Royal FX broad band probe. The interscan delay was set to 5 s, and the number of scans for each sample was 1024. The spectra were phased, baseline-corrected and the chemical shifts were referenced to the peak of hypophosphite at 6.29 ppm. The peaks of phosphate from medium were found at 1.4 ppm and A2P produced a signal at 1.1 ppm. The peaks were integrated, and the molar amounts of phosphate in the medium and A2P were calculated using a known concentration of hypophosphite as a reference. Cumulative A2P release from PTMC5 and PTMC10 was calculated as a percentage of theoretical maximum of A2P in each material when assuming that materials had exactly 5 or 10 wt% of A2P, respectively.

### Scanning electron microscopy

Scanning electron microscopy (SEM) imaging was performed at the Tampere Microscopy Center at Tampere University. SEM imaging was conducted with one donor line of hVSC and hASC after 1 and 7 days of cell culture and samples were fixed and dehydrated as previously described [17, 24]. Briefly, samples were fixed with 5% glutaraldehyde (Sigma-Aldrich) in 0.1 M phosphate buffer (pH 7.4; Sigma-Aldrich). Further, samples were dehydrated with series of increasing ethanol (Altia Oyj, Helsinki, Finland) concentrations and dried with increasing concentration of hexamethyldisilazane (Sigma-Aldrich). After fixing and dehydrating, samples were coated with 5 nm platinum/palladium coating and imaged with Zeiss Crossbeam 540 focused ion beam SEM (Carl Zeiss, Oberkochen, Germany) with 5 kV operating voltage and 40° tilt.

### Cell viability and proliferation

Viability of hVSCs and hASCs was determined after 1 and 7 days of cell culture with LIVE/DEAD Viability/Cytotoxicity kit (L/D, Invitrogen) as previously described [17, 24]. Briefly, samples were incubated at RT for 30 min with solution containing 0.03125 μM of ethidium homodimer-1 and 0.5 μM of calcein acetoxymethyl ester. Samples were imaged with fluorescence microscope (IX51, Olympus, Tokio, Japan).

Cell numbers were measured with CyQUANT Cell Proliferation Assay kit (Invitrogen, Life Technologies) after 1, 7, and 14 days of culture as previously described [16, 17, 24]. The assay was conducted with three donor lines for both hVSCs and hASCs. Three biological replicates were measured with all donor lines (n = 9), and three technical replicates were measured per sample. Cells were lysed with 0.1% Triton X-100 (Sigma-Aldrich), and stored in -70°C. For analysis, CyQUANT GR working solution containing CyQUANT GR dye (400X in DMSO) and cell-lysis buffer (20X) diluted in ultrapure water was added to thawed lysates and measured with Victor NIVO (PerkinElmer, Waltham, Massachusetts, USA) plate reader at 480/530 nm.

### Total collagen content

Sircol Soluble Collagen Assay kit (Biocolor, Carrickfergus, United Kingdom) was used to measure the total collagen content of hVSCs and hASCs after 14 days of cell culture. Analysis was performed with all donor lines with three biological replicates (n = 9), and two technical replicates as previously described [16, 17, 24]. Briefly, samples were incubated in ice-cold 0.5 M acetic acid with 0.1 mg/ml pepsin (Sigma-Aldrich) to extract the collagen. After incubation, samples were collected, Sircol Dye Reagent was added, and samples were mixed with mechanical shaker. Thereafter samples were centrifuged and formed pellets were washed with ice-cold Acid-Salt Wash Reagent. Thereafter, samples were centrifuged, and pellets were resolubilized into Alkali Reagent and measured with Victor NIVO (PerkinElmer) plate reader at 544 nm.

### Quantitative real-time polymerase chain reaction

Quantitative real-time polymerase chain reaction (RT-qPCR) was used to detect the expression of COL I, COL III, α-SMA and elastin in hVSCs and hASCs at day 14 time point as previously described [16, 17, 24]. Messenger ribonucleic acid (mRNA) was isolated from all three donor lines of both cell types with NucleoSpin RNA purification kit (Macherey-Nagel GmbH & Co. KG, Düren, Germany). The cells from one donor were plated onto three replicate materials, and cells were cultured for 14 days. On day 14, the three replicates from the same donor were combined to gain enough mRNA for further analysis. The analysis was repeated with cells from three donors altogether. Isolated mRNA was reverse transcribed to complementary deoxyribonucleic acid (cDNA) using High-Capacity cDNA Reverse Transcriptase Kit (Applied Biosystems, Thermo Fisher Scientific). For the RT-qPCR, cDNA was added to the solution containing SYBR Green PCR Master Mix (Applied Biosystems) and forward and reverse primers [16]. Each sample was run with three technical replicates using QuantStudio 12K Flex System (Applied Biosystems) and results were calculated from technical triplicates of each sample (n = 6–9, samples with technical errors were excluded). Expression of each gene was normalized with the expression of human ribosomal protein lateral stalk subunit (hRPLP0). A presentative cell line from control PS samples was used in normalization. Relative expression was calculated using the mathematical model presented by M.W. Pfaffl. [25]

### Immunocytochemical staining

Immunocytochemical (ICC) staining of α-SMA (ab7817, 1:200, Abcam, Cambridge, UK), COL I (ab138492, 1:2000, Abcam), and vinculin (#700062, 1:100, Invitrogen) were evaluated after culturing hVSCs and hASCs for 7 and 14 days on PTMC0, PTMC5, PTMC10 or PS. Phalloidin (1:500, Sigma-Aldrich) staining was combined with COL I and vinculin staining. Two donor lines from each cell type with two biological replicates were stained in both time points (n = 4). The samples were fixed with 0.2% Triton X-100 (Sigma-Aldrich) in 4% paraformaldehyde (Thermo Fisher Scientific), permeabilized with 1% bovine serum albumin (Sigma-Aldrich), and incubated with above-mentioned primary antibodies overnight at +4°C. The following day, samples were incubated 45 min at +4°C with secondary antibodies (green fluorescence; A-11029, 1:300 or A-21206, 1:400 or A-21206, 1:800, respectively) and phalloidin (red fluorescence). Finally, the cell nuclei were stained with DAPI (blue fluorescence; Sigma-Aldrich), and the samples were imaged with fluorescence microscope (IX51, Olympus).

### Statistical analysis

Statistical analysis was performed with IBM SPSS Statistics (Version 29, IBM Corporation, Armonk, USA). Kruskal-Wallis test with Bonferroni correction was used to evaluate the statistical significance of contact angle (n = 38–40), Sircol (n = 9), CyQUANT (n = 9), and RT-qPCR (n = 6–9) results. Values of *p* < 0.05 were considered significant, and *p* < 0.001 as highly significant. Results were normalized according to the cell culture area on day 7 and 14 time points for CyQUANT and Sircol analyses. Further, the averages of technical replicates were calculated for CyQUANT and Sircol analyses before conducting statistical analyses. For RT-qPCR, statistical analyses were conducted with technical triplicates (n = 6–9) to have enough data for performing statistical analysis.

## Results

### Mechanical properties of ascorbic acid 2-phosphate-releasing poly(trimethylene carbonate)

PTMC containing 0, 5 or 10 wt% of A2P were prepared by co-precipitation in a non-solvent and subsequent drying. After compression molding, the resulting membranes were found to have good handling characteristics and mechanical properties. Elastic moduli (E moduli), yield strengths, and SRSs are given in Table 1.

**Table 1:** Mechanical characteristics of PTMC and A2P-releasing PTMC materials

Material	E modulus (MPa)	Yield strength (MPa)	Suture retention strength (N/mm)
PTMC0	3.9 ± 0.2	1.8 ± 0.1	13.3 ± 3.4
PTMC5	3.4 ± 0.6	1.7 ± 0.2	12.6 ± 0.7
PTMC10	2.6 ± 0.2	1.4 ± 0.1	7.6 ± 0.9

With increasing A2P content in the PTMC, the E modulus, yield strength, and SRS of the materials are somewhat lowered in comparison to pure PTMC. The differences are nevertheless quite small, especially for PTMC5.

### Ascorbic acid 2-phosphate increases the hydrophilicity of the material

**Fig. 1:**
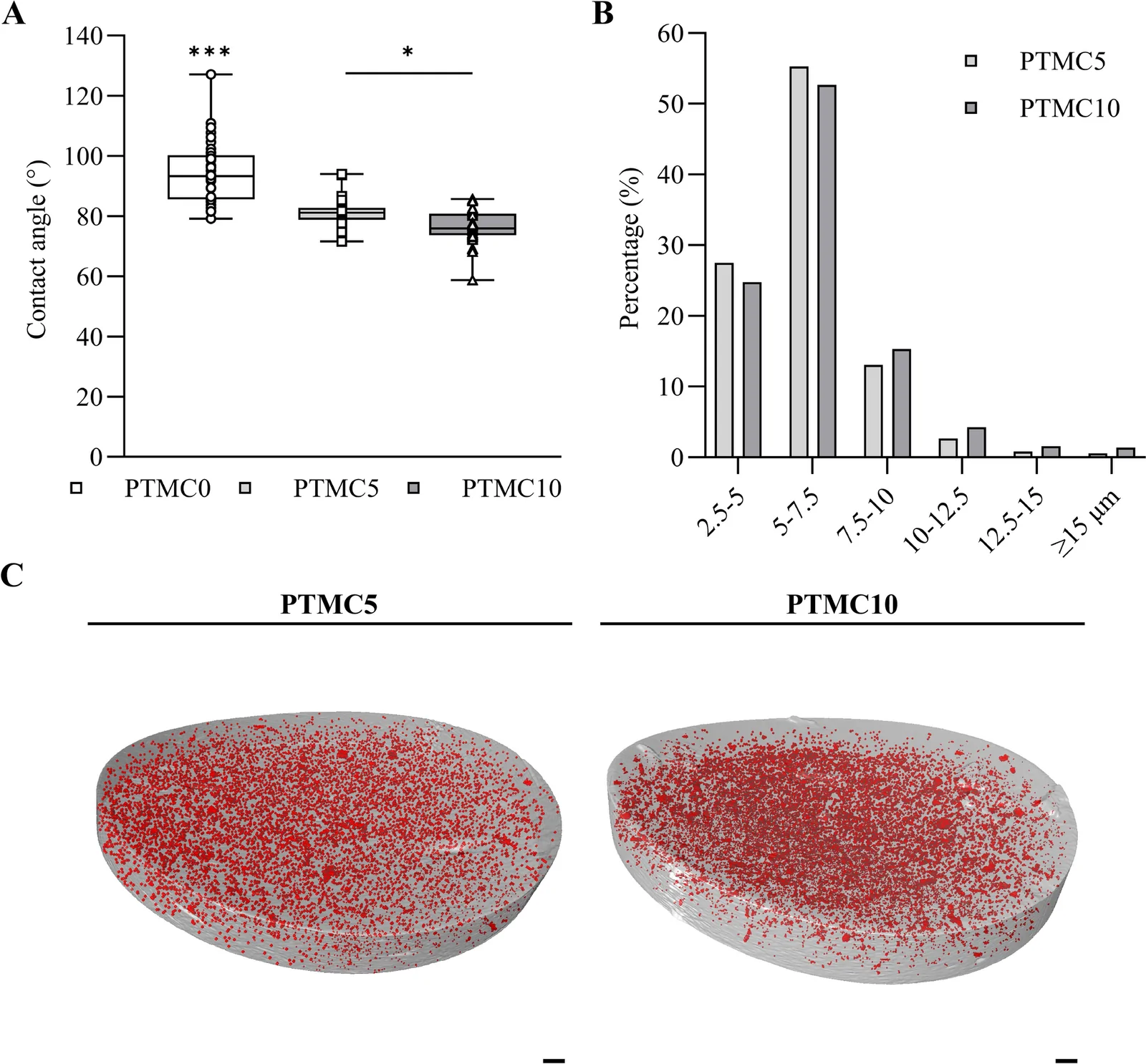
Hydrophilicity and A2P particle size distribution of PTMC materials. **A** The contact angles of PTMC0, PTMC5 and PTMC10, *p < 0.05, ***p < 0.001. **B** A2P particle size distribution in µm and **C** representative micro-CT images showing A2P particles (red) inside PTMC5 and PTMC10 materials, scale bars 100 µm

The hydrophilicity of the different PTMC materials was evaluated with contact angle measurements. Hydrophilicity of PTMC was observed to increase with increasing A2P content. The average contact angles ± SD were 94.2 ± 10.2° for PTMC0, 81.0 ± 4.2° for PTMC5, and 76.4 ± 5.5° for PTMC10 (Fig 1A). The average contact angle was significantly higher on PTMC than on PTMC5 (*p* < 0.001) or PTMC10 (*p* < 0.001). Furthermore, the contact angle was lower on PTMC10 than on PTMC5 (*p* = 0.011).

### Ascorbic acid 2-phosphate particle size distribution in poly(trimethylene carbonate)

A2P particle volume fractions and sizes (Fig. 1B) were assessed with micro-CT imaging from PTMC5 and PTMC10 membranes. A2P particle volume fraction was 0.75 ± 0.34% in PTMC5 and 1.41 ± 0.31% in PTMC10. Most A2P particles within PTMC5 and PTMC10 had diameter of 5–7.5 µm (55.27% and 52.67%, respectively) and only few particles with the diameter greater than 15 µm were found (0.57% and 1.36%, respectively). Visually, A2P particles seemed to be larger and more densely packed in PTMC10 compared to PTMC5 (Fig. 1C).

### Ascorbic acid 2-phosphate release from the membranes

**Fig. 2:**
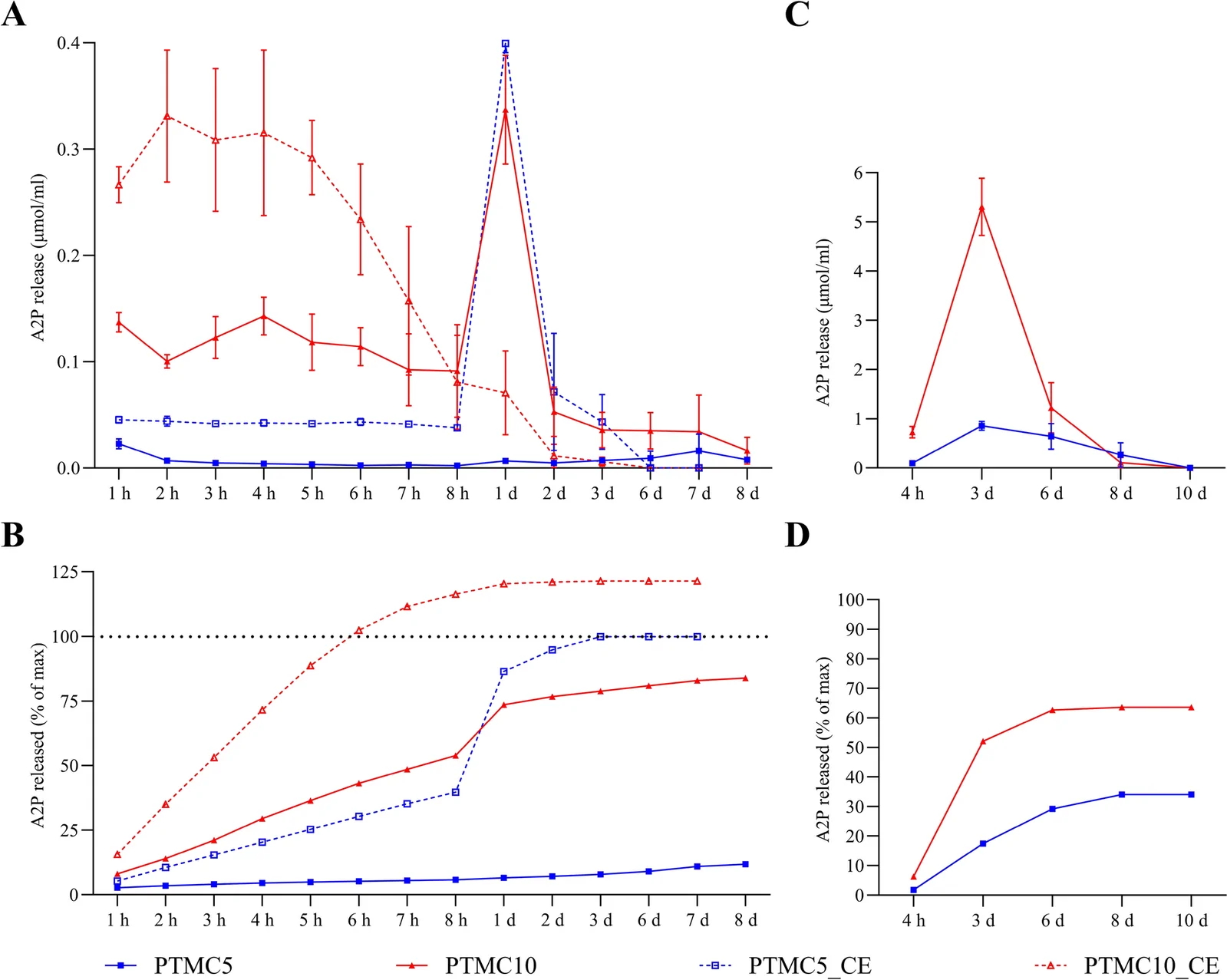
A2P released from PTMC5 and PTMC10 membranes. **A** The amount of A2P released from PTMC5 and PTMC10 membranes in PBS with and without the presence of CE and **B** cumulative release as a percentage of the theoretical maximum in different time points. **C** The amount of A2P released from PTMC5 and PTMC10 in cell culture media in different time points and **D** cumulative release as a percentage of the theoretical maximum

A2P released from PTMC5 and PTMC10 was measured with UV-Vis from samples incubated in PBS with and without CE. The release from PTMC10 in PBS started at a slow and steady rate at the beginning of the incubation but on day 1 the release (0.337 ± 0.051 µmol/ml) had higher peak (Fig. 2A). From PTMC5, release of A2P in PBS occurred in a steady manner with low concentrations. With the presence of CE, A2P was released in a more rapid manner from PTMC10, and the highest release occurred during the first 5 h of incubation. A2P release from PTMC5 with the presence of CE started steadily with low concentrations and peaked at day 1 (0.399 ± 0.009 µmol/ml), after which release continued in small quantities. During the 8-day incubation in PBS, 11.79% and 83.80% of the theoretical maximum were released from PTMC5 and PTMC10, respectively (Fig. 2B). When incubated with CE, 99.89% and 121.42% of the theoretical maximum were released from PTMC5 and PTMC10, respectively.

A2P release in cell culture medium was measured with ^31^P-NMR from 4 h pre-wetting medium and medium samples collected after 3, 6, 8 and 10 days of culture. A2P release was highest between 4 h and day 3 for both PTMC5 (0.857 ± 0.092 µmol/ml) and PTMC10 (5.309 ± 0.583 µmol/ml) (Fig. 2C). After day 3, A2P was still released from the materials, but in smaller quantities. After day 8, A2P was not detected in cell culture medium. The total A2P release from PTMC5 and PTMC10 were 34.02% and 63.63% of the theoretical maximum, respectively (Fig. 2D).

### Ascorbic acid 2-phosphate supports the cell viability and proliferation

**Fig. 3:**
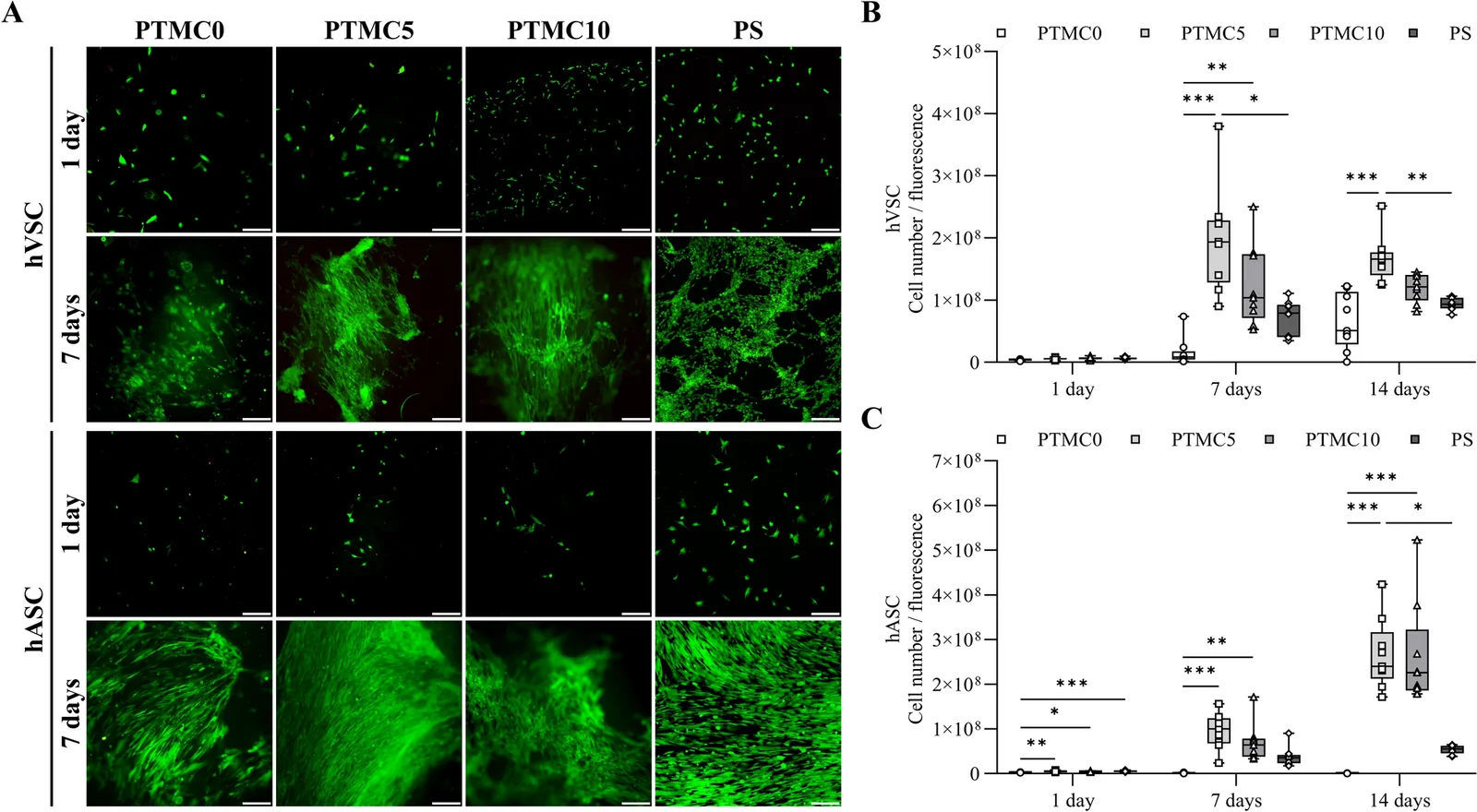
Viability and proliferation of hVSCs and hASCs on PTMC0, PTMC5, PTMC10 and PS. **A** Viable (green) and dead (red) cells on each material, scalebars 200 µm. Proliferation of **B** hVSCs and **C** hASCs on PTMC0, PTMC5, PTMC10 and PS, *p < 0.05, **p < 0.01, ***p < 0.001

The viability of hVSCs and hASCs was evaluated after 1 and 7 days of cell culture on PTMC0, PTMC5, PTMC10, and PS (Fig. 3A) with L/D assay. Both cell types remained viable on all materials, and hardly any dead cells were observed. When visually observed, cell densities seemed to be higher on A2P-releasing materials.

Cell proliferation of hVSCs (Fig. 3B) and hASCs (Fig. 3C) were measured on PTMC0, PTMC5, PTMC10 and PS on days 1, 7, and 14 with CyQUANT assay. Overall, the proliferation of both hVSCs and hASCs were significantly higher on A2P containing membranes. On day 1 there were no significant differences in the hVSC numbers between the different materials. On day 7, the proliferation of hVSCs was significantly higher on PTMC5 compared to PTMC0 (*p* < 0.001) and PS (*p* = 0.038) and the number of hVSCs was also higher on PTMC10 than on PTMC0 (*p* = 0.004). After 14 days of culture, there were more hVSCs on PTMC5 than on PTMC0 (*p* < 0.001) or PS (*p* = 0.001). Furthermore, the number of hVSCs increased significantly during the first 7 days of culturing on PTMC5 (*p* < 0.001), PTMC10 (*p* = 0.002) and PS (*p* = 0.007) plateauing thereafter. On PTMC0, the significant increase in hVSC number was only observed between days 1 and 14 (*p* = 0.002).

On day 1, the number of hASCs on PTMC0 was significantly lower than on PTMC5 (*p* = 0.004), PTMC10 (*p* = 0.018) or PS (*p* = 0.001). On day 7 and 14, the number of hASCs was significantly higher on PTMC5 (*p* < 0.001) and PTMC10 (*p* = 0.001 and *p* < 0.001, respectively) compared to PTMC0. Further, the number of hASCs was higher on PTMC5 than on PS (*p* = 0.020) on day 14. The number of hASCs increased significantly throughout the entire 14-day culture period on A2P-releasing PTMC5 and PTMC10 (*p* < 0.001). On PS, hASCs reached the maximum proliferation on day 7. On PTMC0 there was no significant increase in hASC number during the 14-day culture period and the total number of cells remained low.

### Ascorbic acid 2-phosphate affects the surface topography and cellular adhesion

**Fig. 4:**
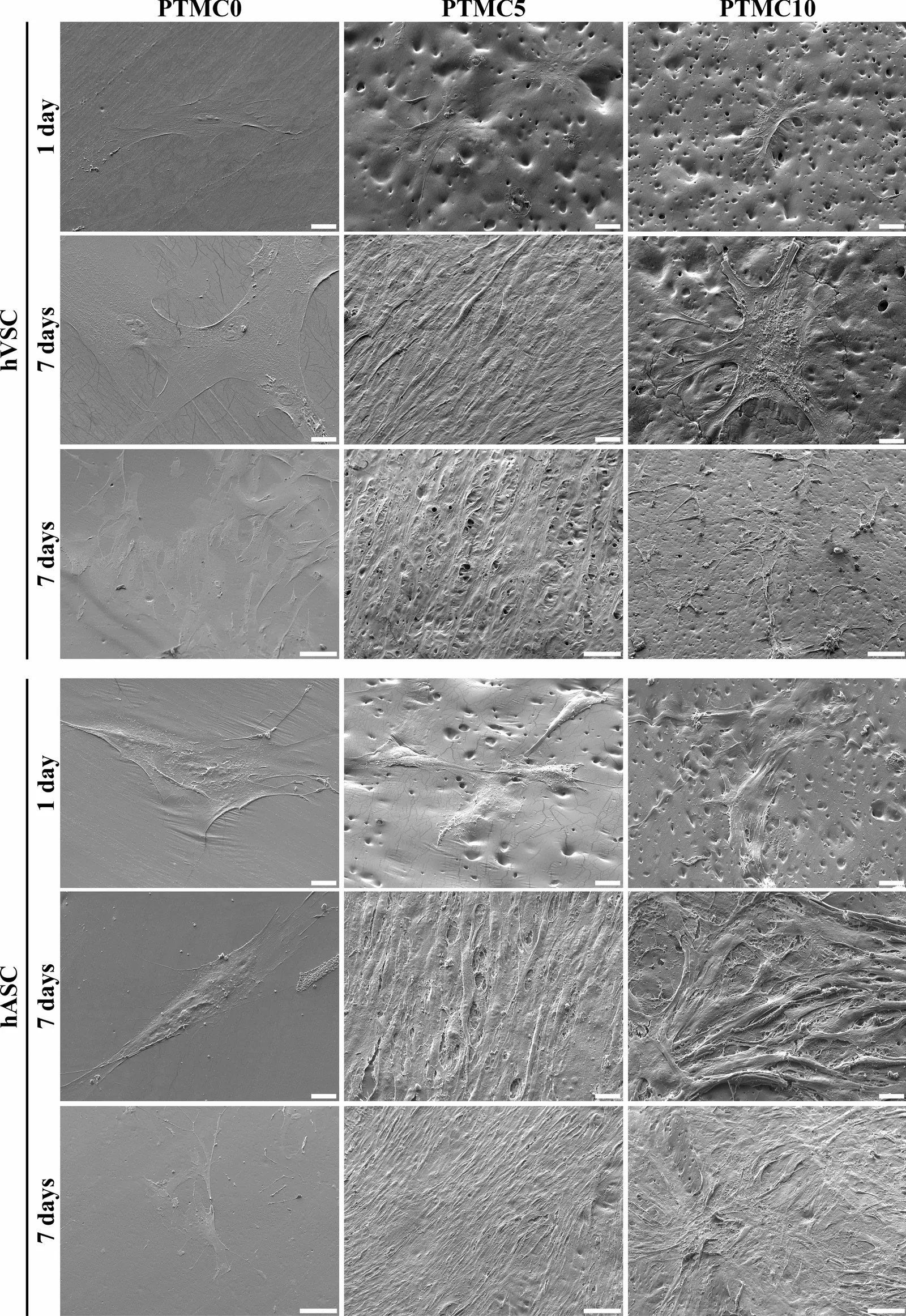
Attachment and morphology of hVSCs and hASCs on PTMC0, PTMC5 and PTMC10. SEM images of the cells on each material on day 1 and 7. Scalebars 20 µm (rows 1–2 and 4–5) and 100 µm (rows 3 and 6)

Surface topography of PTMC membranes and morphology of hVSCs and hASCs were observed with SEM imaging on day 1 and 7 (Fig. 4). SEM imaging showed clear differences in surface structure: PTMC0 had a smooth surface, while the A2P-releasing membranes were rough and porous. On day 1, hVSCs were flattened on PTMC0 surface yet having normal spindle shaped fibroblast morphology on PTMC5 and PTMC10. On day 7, hVSCs remained flat and spread on PTMC0. The hVSCs on PTMC5 had a parallel cell sheet on day 7, whereas some cell clustering was observed on PTMC10. Like hVSCs, the hASCs also demonstrated a flattened morphology on PTMC0 on day 1 and 7. On PTMC5 and PTMC10, hASCs were spindle shaped on day 1 and had organized cell sheets on day 7. According to the SEM images, both cell types seemed to attach better on A2P-releasing PTMC materials and grew following the surface topography of the materials.

**Fig. 5:**
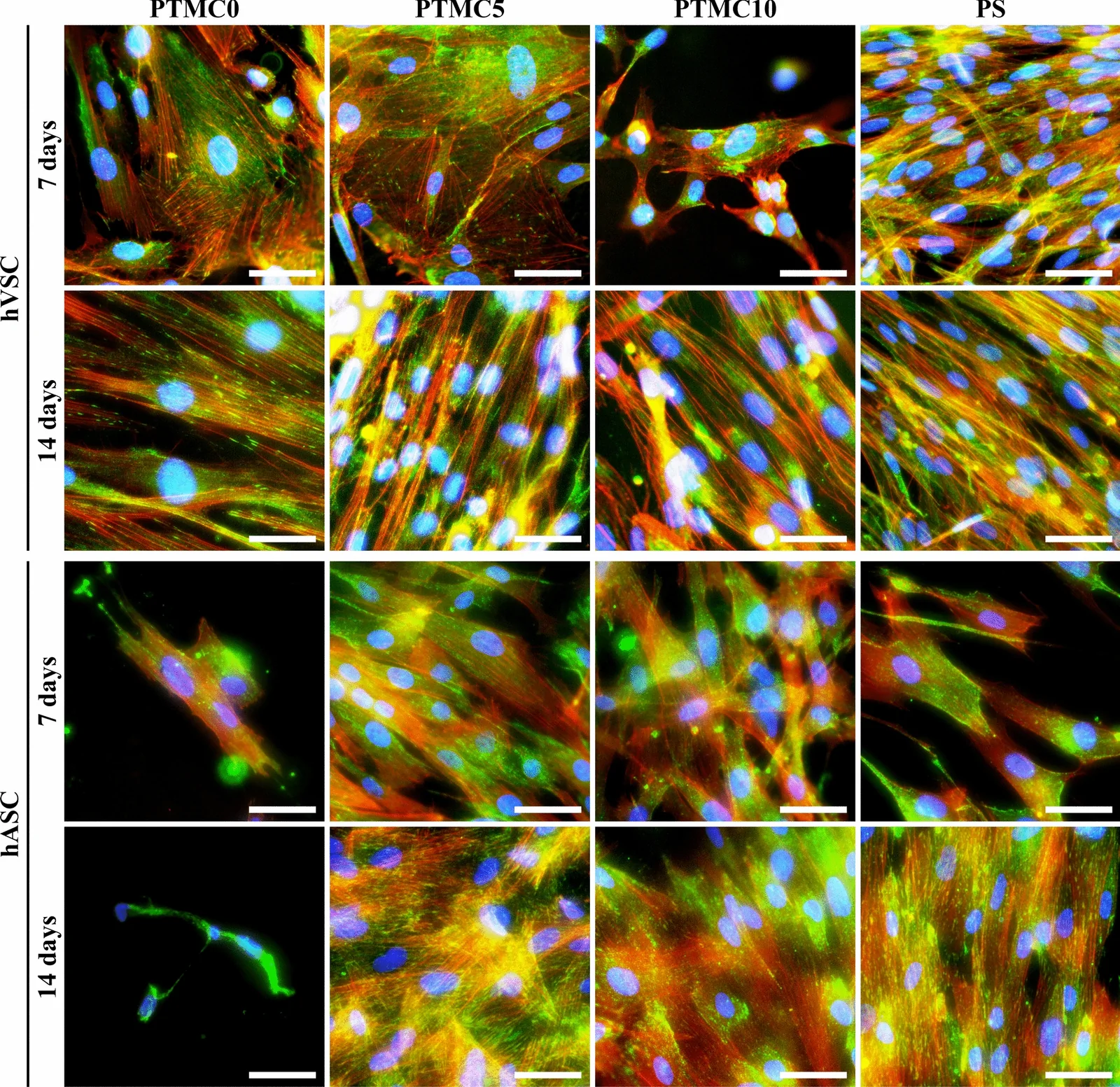
Vinculin staining of hVSCs and hASCs on PTMC0, PTMC5, PTMC10 and PS. Vinculin stained green, actin cytoskeleton red and nuclei blue. Scalebars 50 µm

Attachment of the cells was examined with vinculin staining on days 7 and 14 (Fig. 5). Vinculin was observed in both cell types on all materials. For hVSCs, clear attachment points were observed on all materials except on PTMC10 and for hASCs on all materials except PTMC0.

### Ascorbic acid 2-phosphate increases the collagen production of the cells

**Fig. 6:**
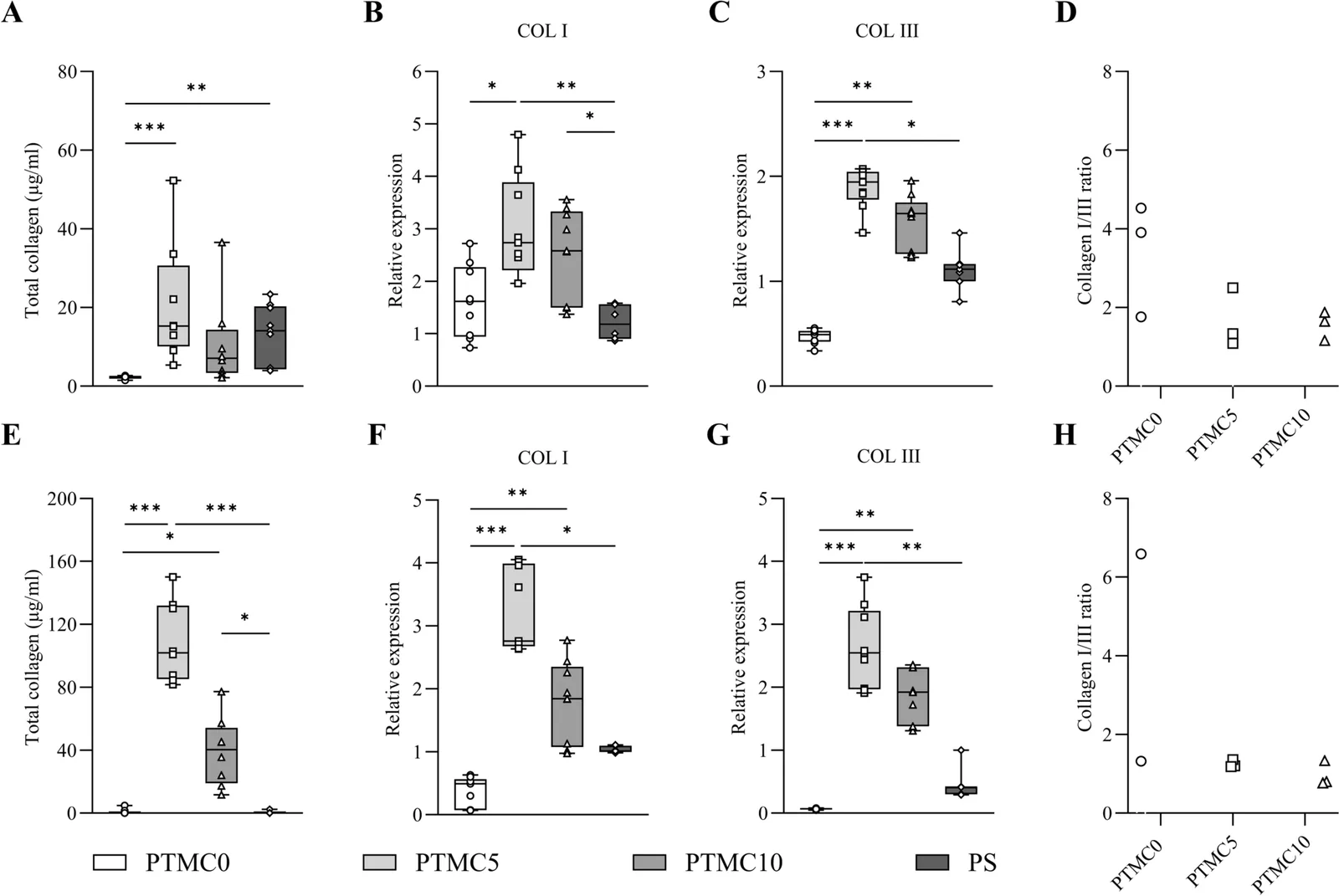
Collagen production of **A–D** hVSCs and **E–H** hASCs on PTMC0, PTMC5, PTMC10 and PS. The total collagen content of **A** hVSCs and **E** hASCs on each material on day 14. The relative COL I expression of **B** hVSCs and **F** hASCs and the relative COL III expression of **C** hVSCs and **G** hASCs on day 14. The ratio of COL I to COL III expression of **D** hVSCs and **H** hASCs on day 14. *p < 0.05, **p < 0.01, ***p < 0.001

The total collagen content produced by hVSCs (Fig. 6A) and hASCs (Fig. 6 E) was analyzed with Sircol assay after 14 days of culturing on PTMC0, PTMC5, PTMC10 and PS. In addition, the expressions of COL I (Fig. 6B, F) and COL III (Fig. 6C, G) were analyzed at day 14 with RT-qPCR. Altogether, A2P increased the total collagen content and COL I and COL III expression of both cell types.

In hVSC cultures, the total amount of collagen was significantly higher on PTMC5 (20.75 ± 15.37 μg/ml) than on PTMC0 (2.27 ± 0.39 μg/ml, *p* < 0.001) (Fig. 6A). However, the collagen content was relatively similar between A2P-releasing PTMC10 (10.69 ± 11.34 μg/ml) and PS (13.26 ± 7.52 μg/ml). Further, the total collagen content of hVSCs on PTMC0 was significantly lower compared to PS (*p* = 0.003). Additionally, hVSCs showed significantly higher expression of COL I on PTMC5 than on PTMC0 (*p* = 0.033) or PS (*p* = 0.004) and COL I expression was significantly higher on PTMC10 compared to PS (*p* = 0.044) (Fig. 6B). The expression of COL I was 1.9 times higher on PTMC5 and 1.6 times higher on PTMC10 compared to PTMC0. Similar results were observed for COL III (Fig. 6C). The expression of COL III was significantly higher on A2P-releasing PTMC5 (*p* < 0.001) and PTMC10 (*p* = 0.002) compared to PTMC0 and significantly higher on PTMC5 compared to PS (*p* = 0.013). The expression of COL III was 4.0 times higher on PTMC5 and 3.3 times higher on PTMC10 compared to PTMC0. Furthermore, the ratio of COL I/III was 3.40 on PTMC0, 1.64 on PTMC5 and 1.56 on PTMC10 (Fig. 6D).

The total collagen content produced in hASC cultures was significantly higher on A2P-releasing materials PTMC5 (108.82 ± 25.57 μg/ml) and PTMC10 (39.26 ± 21.76 μg/ml) compared to PTMC0 (0.72 ± 1.65 μg/ml, *p* < 0.001 and *p* = 0.041, respectively) and PS (0.28 ± 0.83 μg/ml, *p* < 0.001 and *p* = 0.021, respectively) (Fig. 6E). The total amount of collagen produced by hASCs on PTMC0 and PS remained low after 14 days of culture. Furthermore, the expression of COL I was significantly higher on PTMC5 compared to PTMC0 (*p* < 0.001) and PS (*p* = 0.017) and the expression was significantly higher on PTMC10 than on PTMC0 (*p* = 0.006) (Fig. 6F). The expression of COL I was 8.9 times higher on PTMC5 and 4.9 times higher on PTMC10 compared to PTMC0. For COL III, significantly higher expression was observed on PTMC5 than on PTMC0 (*p* < 0.001) or PS (*p* = 0.003), and on PTMC10 compared to PTMC0 (*p* = 0.006) (Fig. 6G). The expression of COL III was 39.9 times higher on PTMC5 and 28.1 times higher on PTMC10 compared to PTMC0. Furthermore, the ratio of COL I/III was 3.96 on PTMC0, 1.25 on PTMC5 and 0.97 on PTMC10 (Fig. 6H)

**Fig. 7:**
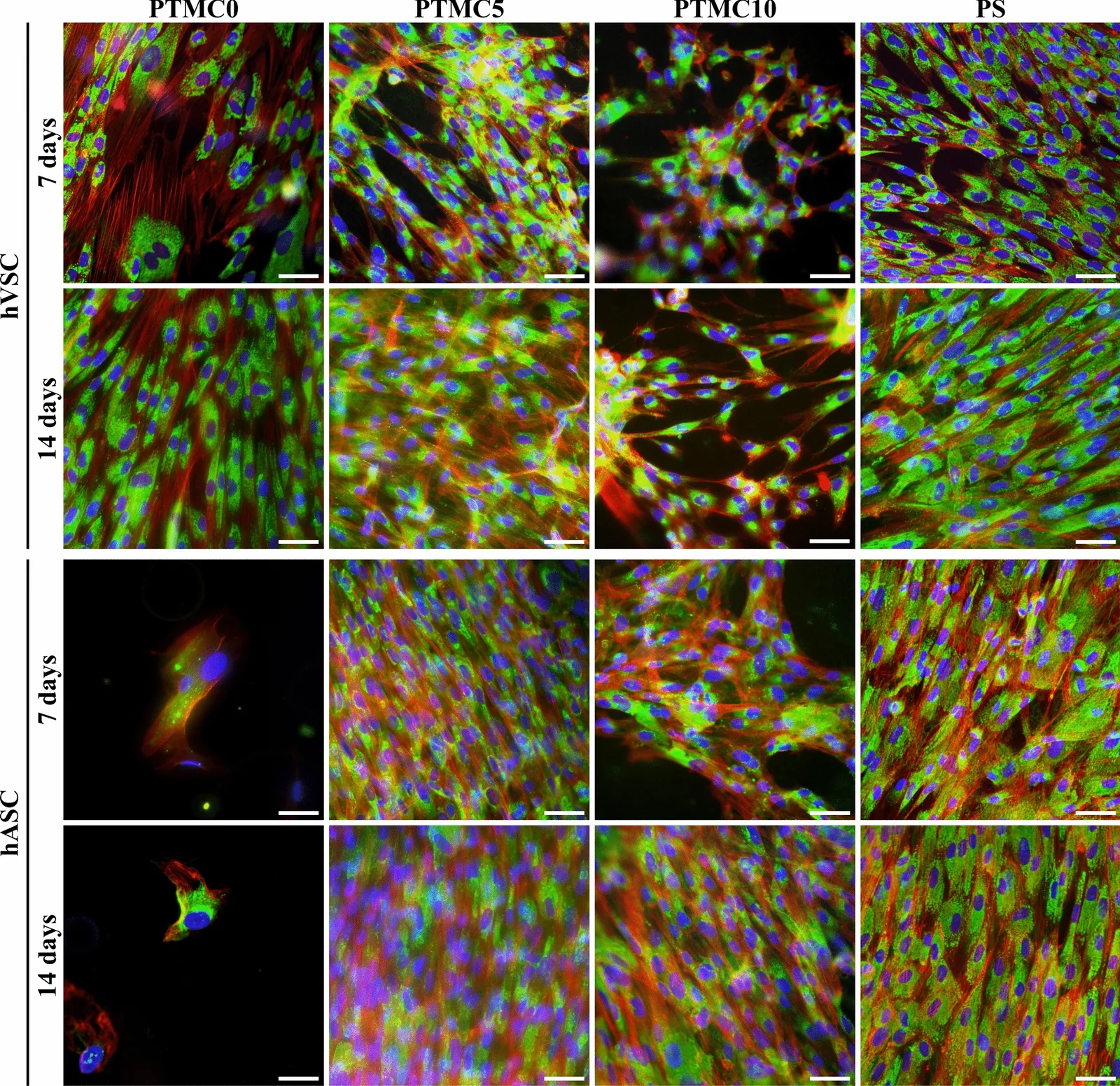
COL I staining of hVSCs and hASCs on PTMC0, PTMC5, PTMC10 and PS. COL I stained green, actin cytoskeleton red and nuclei blue. Scalebars 100 µm

**Fig. 8:**
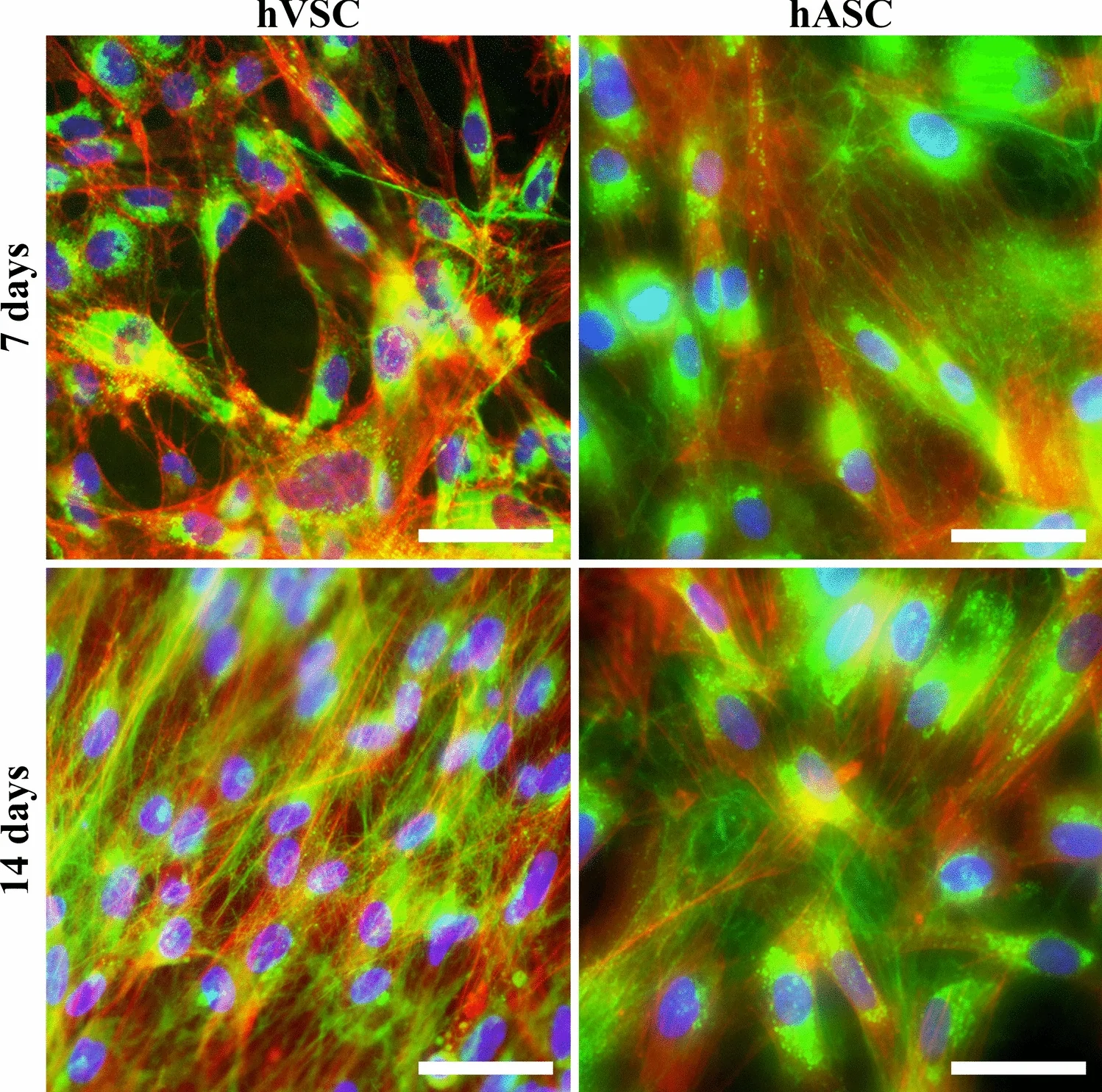
Extracellular COL I fibers in hVSC and hASC cultures on PTMC5. COL I stained green, actin cytoskeleton red and nuclei blue. Scalebars 50 µm

ICC staining of COL I was performed on day 7 and 14 for both hVSCs and hASCs (Fig. 7). On PTMC0, PTMC10, and PS, COL I was observed only inside the cells. Interestingly, on PTMC5 some extracellular collagen fibers were detected on day 7 and 14 with both cell types (Fig. 8).

### Ascorbic acid 2-phosphate increases α-SMA expression of hASCs

**Fig. 9:**
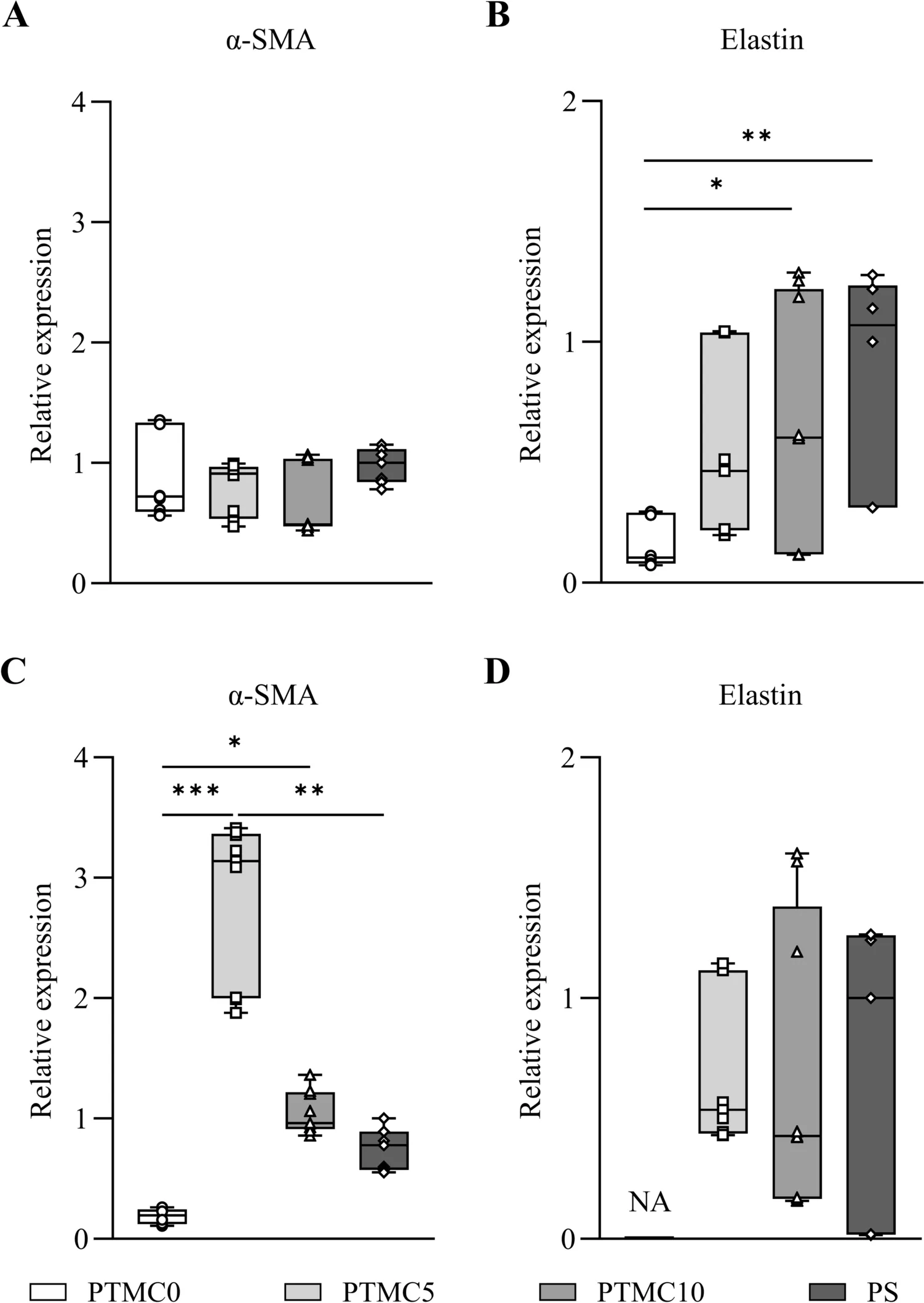
The relative α-SMA and elastin expression of **A–B** hVSCs and **C–D** hASCs on PTMC0, PTMC5, PTMC10 and PS on day 14. *p < 0.05, **p < 0.01, ***p < 0.001

The relative expression of α-SMA and elastin in hVSCs and hASCs were analyzed with RT-qPCR on PTMC0, PTMC5, PTMC10, and PS after 14 days of cell culture (Fig 9). No significant differences were observed in α-SMA expression of hVSCs (Fig 9A). However, the expression of α-SMA in hASCs was significantly increased on A2P-releasing PTMC membranes (Fig 9C). Compared to PTMC0, the expression of α-SMA in hASCs was increased on both PTMC5 (*p* < 0.001) and PTMC10 (*p* = 0.023) and hASCs had significantly higher expression of α-SMA on PTMC5 than on PS (*p* = 0.003). Further, there were no statistical differences in elastin expression in hVSCs between PTMC5, PTMC10, and PS (Fig. 9B). However, the expression of elastin was increased on PTMC10 (*p* = 0.041) and PS (*p* = 0.006) compared to PTMC0. There were no significant differences in elastin expression of hASCs between the materials (Fig. 9C).

**Fig. 10:**
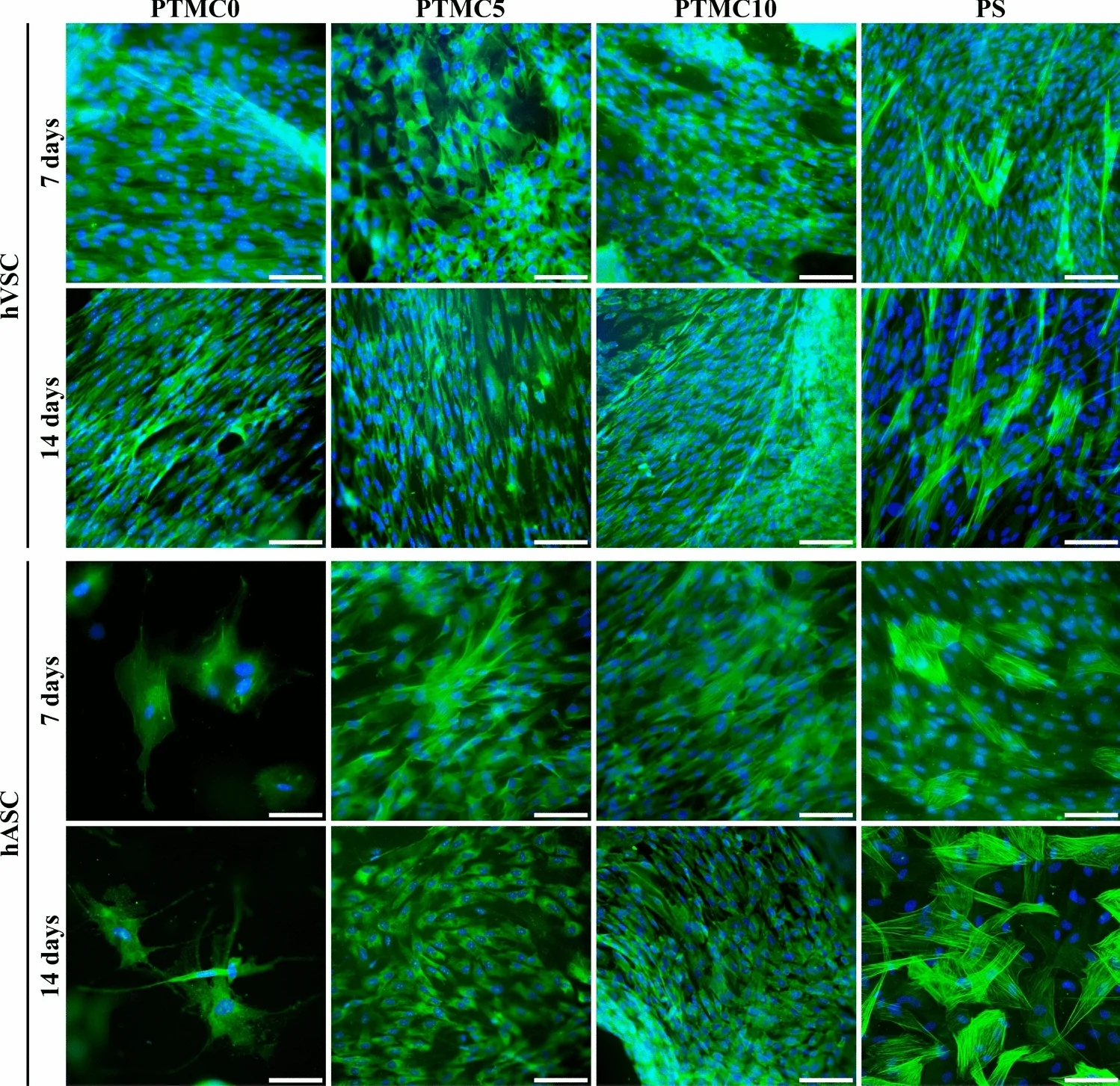
α-SMA staining of hVSCs and hASCs on PTMC0, PTMC5, PTMC10 and PS. α-SMA stained green and nuclei blue. Scalebars 100 µm.

Additionally, staining of α-SMA was observed in both cell types on all the materials (Fig. 10). Some stress fibers were observed on hASCs cultured on PTMC5 and PTMC10 on day 7 and 14 (not shown). Further, stress fibers were detected in both hVSCs and hASCs on PS on day 7 and 14.

## Discussion

Due to the complications associated with non-degradable PP meshes, there is a need for new material options for POP surgery. For the first time, we have manufactured and characterized biodegradable A2P-releasing PTMC materials, and evaluated their impact on hVSC and hASC growth, attachment, and ECM component production. Two different weight percentages of A2P within the same range as in our previous *in vitro* and *in vivo* studies with A2P-releasing PLCL [16, 24, 26] were chosen for this study.

All PTMC materials had good handling characteristics and mechanical properties. The determined values for the E modulus and yield strength of PTMC materials used in this study are indicative of an elastic rubber-like material and in accordance with previous determinations [6]. It is essential that biomaterials intended for implantation exhibit mechanical properties matching those of the native tissue at the implantation site since high stiffness of the material has been proposed to be one factor causing complications with PP meshes used in POP repair. [27, 28] Therefore, using more elastic materials that possess mechanical properties closer to the native tissue might help to prevent mesh-related problems. Reported values for the ultimate tensile strength and E modulus of vaginal tissues have variation depending on the donor age, POP stage, and sample processing. Ultimate tensile strength (UTS) of non-POP and POP tissues vary approximately between 0.39–3.9 and 0.24–5.8 MPa, respectively. [14, 29] Yield strengths of PTMC materials tested in this study were 1.3–1.9 MPa, which fall within the range of reported values for vaginal tissues. However, UTS and yield strength are not completely comparable to each other. Further, E moduli of non-POP and POP tissues have been reported to vary between 5.17–11.5 and 8.75–13.9 MPa, respectively [14, 29]. In this study, E moduli of PTMC materials were 2.4–4.1 MPa.

*In vitro* release measurements showed that A2P release from PTMC occurs in a controlled manner. The total amounts of A2P released in PBS were only 11.79% and 83.80% from the theoretical maximum from PTMC5 and PTMC10, respectively. We demonstrated that A2P release from PTMC into PBS occurred at a slow rate, differing from quick A2P release reported previously from PLA and PLCL [15, 16]. Previously reported quick release from PLA and PLCL into PBS is most probably due to their degradation via hydrolytic bulk erosion [30]. In this study, in the presence of CE, A2P release from PTMC was more rapid, and the total release was 99.89% and 121.41% from the theoretical maximum from PTMC5 and PTMC10, respectively. Differences observed in the A2P release profile from PTMC under enzymatic and non-enzymatic conditions are most likely attributed by the enzymatic surface erosion of PTMC [9]. However, it has not been demonstrated whether controlled or quick release of A2P would be more beneficial for connective tissue remodeling. Even though the presence of CE better mimics the enzymatic *in vivo* conditions compared to plain PBS, there are other possible factors, for example, immune cell activity and oxidation, which has been proposed to affect PTMC degradation *in vivo* [31]. Therefore, more research is needed in the future to evaluate how A2P is released from PTMC *in vivo*. Further, the total release from PTMC10 in the presence of CE was measured to be over 100% from the theoretical maximum which suggests that A2P is not completely evenly distributed within the materials. Further, micro-CT analysis revealed that A2P particle sizes within the materials were similar but some heterogeneity in A2P particle distribution was observed in PTMC10. Heterogeneity might affect the cell response locally, since more A2P can be released from areas with higher particle density. In the future, more research is needed to optimize the material preparation and especially the distribution of A2P within the material. Further, we have devised a fast and convenient method for quantification of A2P directly from the culture medium via ^31^P-NMR. NMR measurements showed that the highest A2P release from the membranes occurred within the very first days of cell culture, however, the release continued for several days after that. Further, the total A2P measured from the culture medium from PTMC5 and PTMC10 were 34.02% and 63.63% of the theoretical maximum, respectively, and no A2P was detected after day 8. This might be because the material does not have sufficient time to degrade under cell culture conditions within 14 days to release all A2P, or the cells have utilized some of the A2P released in the medium within the culture period [21].

Hydrophilicity of the material has been demonstrated to improve cell-material interactions by increasing the attachment, spreading, and proliferation of the cells [31, 32]. Contact angle measurements conducted in this study show that A2P significantly increases the hydrophilicity of the PTMC as previously demonstrated with A2P containing PLA and PLCL [15, 16]. Therefore, increased hydrophilicity could be one factor explaining the increase in cellular attachment and proliferation on A2P-releasing PTMC. Further, the surface topography of the material has been previously demonstrated to affect the attachment of cells [33, 34]. Although no quantitative measurements on surface roughness were performed, qualitative assessment of the SEM images showed that the release of A2P increases the roughness of the PTMC surface while pure PTMC has a totally smooth surface. Further, both cell types had flat morphology on pure PTMC and regular spindle shaped morphology with cell sheet formation on PTMC5. Interestingly, on PTMC10 we observed cluster formation for hVSCs in SEM and ICC stainings. However, hASCs seemed to have natural spindle shaped morphology and formed cell sheets on both PTMC5 and PTMC10. Cell clustering might be related to cellular attachment and the surface properties, such as hydrophilicity or surface topography, of the material [31–34]. Similar cluster formation has been observed in previous studies with hVSCs and hASCs cultured on PLCL [22, 24]. However, more thorough research is needed to estimate if A2P itself affects cell morphology and clustering. Further, in vinculin stainings, hVSCs exhibited distinct attachment points on PTMC0 and PTMC5 whereas hASCs showed attachment points on both A2P-releasing materials, which might be related to the cell type-specific response for material topography or hydrophilicity or both. Additionally, it has been previously demonstrated that fibroblasts tend to have better attachment on smoother surfaces [35] and hASCs on rougher surfaces [36] which corresponds with our findings.

The viability of hVSCs and hASCs on all PTMC-based membranes indicates the good biocompatibility of the PTMC, which has also been demonstrated in previous studies with different cell types [8, 37, 38]. However, this is the first study to investigate A2P-releasing PTMC material and our findings show that A2P increased the proliferation of both hVSCs and hASCs as previously demonstrated by Mangir *et al.* with human dermal fibroblasts cultured on A2P-releasing PLA and Kurki *et al.* with human vaginal fibroblasts (i.e. hVSCs) and hASCs cultured on A2P-releasing PLCL [15, 16]. For hASCs, significant increase in proliferation was observed on both PTMC5 and PTMC10 during the whole 14-day culture period even though A2P was not detected from the culture medium after day 8. For hVSCs significant increase in proliferation was observed between day 1 and 7 on PTMC5 and PTMC10. The highest proliferation was measured on PTMC5 with both cell types, which indicates that A2P increases the proliferation in a dose–dependent manner as demonstrated previously with AA and A2P [39–41]. Despite limited research on A2P-releasing materials, AA- or A2P-supplemented medium has been shown to enhance proliferation of hASCs [20, 41] and fibroblasts [42, 43]. However, the cytotoxic effect of AA via H_2_O_2_ generation has been demonstrated with cancer cells when AA concentration exceeds 3 mM [44]. In our study, A2P concentration measured from the cell culture medium between 4 h and day 3 from PTMC10 was over 5 mM which could explain why cells proliferated more on PTMC5 compared to PTMC10.

For POP applications, supporting the ECM production and tissue remodeling of connective tissues is essential. The predominant ECM components in the pelvic floor connective tissues are COL I, COL III and elastin [12, 13]. Previous studies with hASCs and hVSCs have demonstrated that A2P increases the total collagen production of these cells [16, 17, 20, 24] supporting the results of this study. The total collagen production of hVSCs and hASCs were measured to be higher on A2P-releasing PTMC materials than on PTMC0. For hVSCs, some amount of collagen was measured on all materials, which is most probably due to the fibroblasts’ innate ability to produce collagen [13]. However, the results show that the collagen production of hVSCs was higher on A2P-releasing PTMC5 than on other materials. On the other hand, A2P increased the collagen production of hASCs on both PTMC5 and PTMC10, and almost no collagen was measured on PTMC0 or PS. We observed that A2P seems to affect the collagen production of the cells in dose-dependent manner implying that PTMC with 5 wt% of A2P would be best to support the collagen production, even though the difference was not statistically significant compared to PTMC10. Similar dose-dependent effect of AA and A2P on collagen production have also been reported previously [45, 46]. However, extracellular COL I fibers were only observed on PTMC5 with both cell types, which indicates promoted collagen maturation on PTMC5.

COL I and III are essential components of pelvic floor connective tissues. Our results show that A2P increased the gene expression of COL I as previously demonstrated [17, 20, 21]. COL I expression was 1.9 times higher on PTMC5 and 1.5 times higher on PTMC10 with hVSCs, and 8.9 times higher on PTMC5 and 4.9 times higher on PTMC10 with hASCs compared to PTMC0. Previous studies have shown that the pelvic floor fascia of POP patients has decreased levels of COL I which is responsible for providing mechanical strength to the tissues [47, 48]. We demonstrated that A2P-releasing PTMC increases the COL I synthesis which could improve the mechanical properties of novel tissue formed on the pelvic floor. Further, COL III expression was also increased on A2P-releasing PTMC materials with both cell types. Similar results have been previously reported with different cell types [24, 49]. Research on COL III content of POP tissues has been conflicting. Some studies have concluded that COL III expression is increased in the connective tissues of POP patients [48, 50] whereas some studies have reported decreased COL III expression [51, 52]. However, in our study the effect on COL I expression was more evident than the increase in COL III expression. Previous research also suggests that COL I may have a more significant role in connective tissue strength and POP development than COL III, yet no optimal ratio of COL I to COL III has been identified [53].

Mechanical properties of pelvic floor connective tissues are also dependent on their elastin content since elastin increases the flexibility of connective tissues and allows them to return to their original shape after stretching [12]. Decreased synthesis and increased degradation of elastin has been reported in the connective tissues of POP patients [12, 47]. In the current study, there were no significant differences in elastin expression of hASCs on different PTMC materials which indicates that A2P did not affect the elastin expression of the cells. Similar results have been reported previously with a porous PLCL 3D scaffold containing 8 wt% of A2P [24]. However, in another study, 7 wt% of A2P was found to increase the elastin expression of hASCs on perforated PLCL [16]. Further, in the current study, elastin expression of hVSCs was increased on PTMC10 and PS compared to PTMC0. Previous research on how A2P affects the elastin production of cells has been conflicting. Similar to our results, A2P was previously reported to increase the elastin expression of hVSCs on A2P containing PLCL [16]. However, another study using A2P containing PLCL reported that there was no significant increase in elastin expression of hVSCs [17]. With other cell types, AA as medium supplement has been shown to have no effect on elastin synthesis [54], or to decrease it in time- and dose-dependent manner [55], or to even increase it in long-term exposure [56]. Therefore, further research is needed to demonstrate the effect of A2P on elastin expression of different cell types.

Connective tissue remodeling occurs when fibroblasts differentiate into myofibroblasts expressing α-SMA and secreting ECM components [57, 58]. In our study, α-SMA expression of hASCs was increased on PTMC materials releasing A2P as demonstrated in previous research with A2P-releasing PLCL [16, 24]. In previous studies, increased expression of α-SMA combined with elevated collagen production and formation of stress fibers has been associated with myofibroblastic differentiation of hASCs [59]. In this study, A2P increased the expression of α-SMA, collagen and stress fibers in hASCs, which suggests the differentiation of hASCs towards a myofibroblastic direction. Increased myofibroblastic activity could help to increase connective tissue remodeling in the pelvic floor [13]. However, excessive myofibroblast activity can also lead to fibrosis development [13, 60]. Further *in vivo* research is needed to evaluate whether A2P-releasing PTMC increases natural connective tissue remodeling or fibrosis formation. Expression of α-SMA was detected in hVSCs but A2P did not increase the expression. In previous research, POP patient derived hVSCs showed increased α-SMA expression compared to hVSCs from non-POP patients. In addition, stiffness of the cell environment has been shown to increase the α-SMA production of hVSCs. [57] This suggests that hVSCs naturally express the α-SMA, but increased expression might be related to the mechanical properties of the cell environment. Both cell types expressed α-SMA in ICC stainings, but α-SMA stress fibers were observed mainly on PS which might be due to the material properties since previous research indicates that stress fibers are formed when cells are cultured on stiffer materials [58].

In conclusion, we demonstrated that novel A2P-releasing PTMC materials have promising properties for POP tissue engineering. All PTMC membranes supported the viability of the hVSCs and hASCs. Particularly, A2P increased the proliferation of both cell types, and ideal cellular attachment and cell sheet formation was observed on PTMC5. Further, the total collagen production and expression of COL I and COL III were increased on PTMC5 membranes with both cell types.

## Data Availability

The data presented in this study are available on request from all authors.
